# Sperm Metabolomics through Nuclear Magnetic Resonance Spectroscopy

**DOI:** 10.3390/ani11061669

**Published:** 2021-06-03

**Authors:** Marta Lombó, Sara Ruiz-Díaz, Alfonso Gutiérrez-Adán, María-Jesús Sánchez-Calabuig

**Affiliations:** 1 Department of Animal Reproduction, INIA, Av. Puerta de Hierro, 18, 28040 Madrid, Spain; marta.lombo@inia.es (M.L.); sara.rd.1992@gmail.com (S.R.-D.); agutierr@inia.es (A.G.-A.); 2 Mistral Fertility Clinics S.L., Clínica Tambre, 28002 Madrid, Spain; 3 Department of Animal Medicine and Surgery, Faculty of Veterinary Science, University Complutense of Madrid, 28040 Madrid, Spain

**Keywords:** NMR, male fertility, sperm metabolites, mammalian species

## Abstract

**Simple Summary:**

Proton nuclear magnetic resonance spectroscopy (^1^ H-NMR) is of special interest for the analysis of metabolites present in seminal plasma and spermatozoa. This metabolomic approach has been used to identify the presence of new biomarkers or their proportions in a non-invasive manner and is, therefore, an interesting tool for male fertility diagnosis. In this paper, we review current knowledge of the use of ^1^ H-NMR to examine sperm metabolomics in different species with special attention paid to humans and farm animals. We also describe the use of ^1^ H-NMR to establish a possible relationship between the mammalian diet and the presence of certain hydrophilic and lipophilic metabolites in spermatozoa.

**Abstract:**

This report reviews current knowledge of sperm metabolomics analysis using proton nuclear magnetic resonance spectroscopy (^1^ H-NMR) with particular emphasis on human and farm animals. First, we present the benefits of NMR over other techniques to identify sperm metabolites and then describe the specific methodology required for NMR sperm analysis, stressing the importance of analyzing metabolites extracted from both the hydrophilic and lipophilic phases. This is followed by a description of advances produced to date in the use of NMR to diagnose infertility in humans and to identify metabolic differences among the sperm of mammalian herbivore, carnivore, and omnivore species. This last application of NMR mainly seeks to explore the possible use of lipids to fuel sperm physiology, contrary to previous theories that glycolysis and oxidative phosphorylation (OXPHOS) are the only sources of sperm energy. This review describes the use of NMR to identify sperm and seminal plasma metabolites as possible indicators of semen quality, and to examine the metabolites needed to maintain sperm motility, induce their capacitation, and consequently, to predict animal fertility.

## 1. Introduction

The field of reproductive biology has recently undergone a series of advances in the form of high-throughput massive molecular tools, collectively known as OMICs, for the analysis of biological samples. This new era of omics consists of the study of genes (genomics), mRNA (transcriptomics), proteins (proteomics), epigenetic marks (epigenomics) and metabolites (metabolomics) [1]. Metabolomics is the assessment of all the metabolites (low molecular weight molecules of under 1 kDa arising from metabolic pathways) of a biological system (secretions, cells, tissues and organs), including amino acids, lipids, carbohydrates, nucleotides and hormones [2]. To define a cell phenotype, metabolomics is thought to be more accurate than transcriptomics or proteomics as the metabolites present in a cell provide information on other processes occurring downstream from gene expression and mRNA translation [3].

Metabolomics has been recently proposed as useful to predict male infertility [4,5]. So far, techniques used in routine clinical practice to determine semen quality have relied on microscopy for sperm counts and morphology and motility tests [6]. Despite their widespread use since they were first developed by Macleod [7], these tests are usually inadequate due to observer bias and lack of standardization across the different laboratories [8]. Additionally, in the farming industry, semen analysis needs to be done after puberty so raising potentially subfertile or infertile males can cause economic and time losses [3]. If we consider that infertility currently affects 10 to 15% of men of reproductive age [9] and that over 70% of livestock and poultry are produced by artificial insemination [10], there is a need for non-biased and non-destructive methods of sperm assessment that can be performed earlier in the life of a male. In this context, it has been proposed that the metabolites of both sperm and seminal plasma could be biomarkers of infertility in men and in male farm animals [11,12,13].

The use of metabolomics to predict male fertility is supported by the observation that spermatozoa metabolize a wide range of substances that are somehow related to the signaling pathways implicated in their motility, capacitation, hyperactivation and acrosome reaction [14,15]. Indeed, spermatozoa produce a great deal of ATP, most of which is consumed by axonemal dynein to promote sperm motility, whereas the rest is needed for sperm capacitation [16,17]. In sperm cells, ATP is generated through two main processes: glycolysis and mitochondrial oxidative phosphorylation (OXPHOS) [18,19]. Glycolytic enzymes occur in the main sperm midpiece bound to the fibrous sheath of the flagellum where they can easily provide the flagellar filaments with energy [20]. In contrast, OXPHOS requires that the ATP produced in the tightly packed mitochondria of the midpiece quickly diffuses to the distal end of the flagellum [21]. Although in species such as sea urchin and bull, ATP diffusion from the midpiece to flagellum was found sufficient to sustain motility [22], this seems an unlikely mechanism for the large sperm of rodents [20]. Moreover, glycolysis is much less energetically efficient than OXPHOS (it yields 2 versus 30 ATP molecules) so it is usually restricted to hypoxic conditions or when there is an abundance of glycolytic substrates. So, while these two mechanisms are closely related, the different species have adapted to select one or the other as their major ATP source depending on the oxygen conditions and substrates available [18]. Moreover, in some species, lipid metabolism has been attributed a role in sperm energy production [23] and it has been proposed that human sperm motility can be also fueled by the oxidation of endogenous lipids [23,24]. While both glucose and fructose have been found to be metabolized by spermatozoa in the domestic cat (*Felis catus*), cheetah (*Acinonyx jubatus*) and clouded leopard (*Neofelis nebulosa*), it seems that endogenous lipids provide the energy to support sperm motility in these species [25]. Collectively, the available data indicate that sperm metabolism is species-specific and besides depending on motility patterns and sperm lifespan in the female genital tract, they also reflect adaptations to the availability of substrates in the corresponding environment. Hence, knowledge of the sperm metabolic profile is important both to find biomarkers able to predict sperm quality and fertility and to define the specific components of sperm storage, capacitation and fertilization media required by each species.

## 2. NMR Applied to Sperm Analysis

Since first developed in the mid-twentieth century, nuclear magnetic resonance (NMR) has been used in the study of organs, tissues and cells from a biochemical perspective [26,27,28]. Today, NMR is one of the most powerful techniques to identify metabolites, providing researchers with more accurate information on cell metabolism [29]. The first MNR studies of sperm metabolites were carried out in the goat [30]. From this moment onwards, many sperm metabolites have been identified in several species [25,31,32,33]. Below we briefly describe the protocol, including several critical points, that should be followed when analyzing sperm cells.

Semen samples should be washed with a sperm medium and then centrifuged by density gradient to purify the live sperm. The purity of sperm samples should be checked by phase contrast microscopy to identify samples with no detectable contamination with non-sperm cells after processing. Next, sperm are washed three times with cold PBS, frozen in liquid nitrogen and stored at −80 °C for further metabolite extraction, although metabolites can be directly extracted and lyophilized. Due to the relatively low sensitivity of NMR, a high number of spermatozoa is needed to provide a sufficient amount of metabolites. The sperm pellet must be isolated and sperm concentration adjusted to ~150–200 million motile sperm cells.

There are several protocols for metabolite extraction from sperm [33,34,35]. Here, we describe how to extract hydrophilic and lipophilic products from sperm cells. To extract low-molecular weight metabolites, the sperm cell pellets should be pre-treated by methanol extraction. Samples are first defrosted at room temperature (RT) for 5 min slowly in ice. Next, 1.3 mL of CHCl_3_:MeOH:ddH_2_O at a ratio of 41.7:35.6:32.7 (*v*/*v*/*v*) are added to each sperm sample, which are then stored at 4 °C with agitation for 4 h. The mixture is then centrifuged at 4 °C at max-speed (~25.000–30.000× *g*) for 30 min. The upper and lower phases of the dissolution (hydrophilic and lipophilic phases respectively) are separated into two 2 mL Eppendorf tubes. All the samples are then dried in a SpeedVac (Thermo Fisher Scientific, Waltham, MA, USA). To prepare hydrophilic samples for NMR, the lyophilized product is re-suspended in 500 μL of deuterium oxide (D_2_O) and 0.11 μM of DSS (sodium trimethylsilylpropanesulfonate). Finally, after a brief vortex, 500 μL of the sperm extract is pipetted into a 5 mm NMR tube. Lipophilic extracts are resuspended in 300 μL of DMSO-d6 with 4 mM of TPP (triphenylphosphine) and transferred to a Shigemi tube.

Once extracted, the metabolites can be analyzed. For each sample, 1-dimensional (1 D) and 2-dimensional (2 D) ^1^ H (proton) NMR spectra can be obtained using a Bruker spectrometer (AVANCE III, Bruker Biospin GmbH, Reinsthetten, Germany) equipped with a ^1^ H cryoprobe with a z-gradient and automatic tuning and matching unit measured at 298 K in an 800 MHz. The background of the water content can be minimized by solvent suppression or using WATERGATE (WATER suppression by Gradient Tailored Excitation). As the sample once loaded creates an imbalance in the magnetic field, shim coils should be used to increase magnetic field homogeneity by adjusting the current that flows through the sample (a process known as shimming). 1 D ^1^ H-NMR can be collected using a Carr–Purcell–Meiboom–Gill (CPMG) pulse sequence, whereas data analysis can be done using the TopSpin 3.5 software (Bruker Biospin GmbH, Reinsthetten, Germany). First, through Fourier transformation, a signal is converted from the time domain to the frequency domain, which allows for the determination of the molecular structure at the atomic level in an aqueous solution. Both spectra and baseline are automatically corrected, referenced to the peak of a chemical shift standard compound and calibrated to 0 ppm for further analysis. The absolute integrals are obtained as the sum of the values below the peak. Next, to quantify the peaks of each metabolite, these numbers are divided by the integral of the standard compound peak. Eventually, metabolite peaks are normalized by dividing their peak integrals by the integral value of the whole chemical shift observed within the visible spectrum.

Currently, NMR, along with liquid chromatography coupled to single-stage mass spectrometry (LC–MS), gas chromatography coupled to single-stage mass spectrometry (GC–MS), and tandem mass spectrometry (LC–MS/MS), are the most commonly used techniques in metabolomics. Although in most cases these techniques are complementary [33,36], in the past two decades, NMR has been increasingly employed to assess the presence of metabolites because it has several advantages over both LC–MS and GC–MS [37]. NMR is a high-throughput, non-destructive and non-biased method and so is very useful for in vivo and large-scale studies as well as fluxomics (the study of metabolic fluxes in a cell). On the contrary, LC–MS and GC–MS are destructive methods, so they are worthless for examining living samples. Further, the results provided by NMR are exceedingly reproducible and can be easily quantified [38]. Protein-bound metabolites, such as lipoproteins, as well as inorganic metabolites and ions can be analyzed by this method, but are not detected either by LC–MS or GC–MS [39]. Moreover, NMR is able to detect around 50–200 metabolites at concentrations higher than 1 μM, whereas LC–MS identifies over 1000 metabolites at concentrations between 10 and 100 nM. Notwithstanding, improvements in the sensitivity of NMR are underway in terms of design, pulse sequences, and magnet field strength [37].

## 3. Human Sperm Metabolites

While sperm metabolites in species such as the goat were identified more than twenty years ago [30], human sperm metabolites have been only recently characterized [11,33,40]. Firstly, Paiva and colleagues [33] were able to detect 69 metabolites in human sperm extracts, 42 of them by NMR and the remaining 27 by GC–MS. The dominant metabolites detected by ^1^ H-NMR mainly belonged to four categories: aminoacids and peptides (alanine, arginine, creatine, glutamine, N-acetyl tyrosine, tyrosine, and valine), lipids (acetylcarnitine, glycerophosphocholine (GPC), butyrate, caprate, caprylate, 2-methyl glutarate and 2-hydroxy-3-methylvalerate), organic acids and derivatives (lactate, acetate, formate, glycolate, isobutyrate, azelate, 3-hydroxyisobutyrate, 2-oxoglutarate; 3-hydroxybutyrate) and aliphatic acyclic compounds (phosphocholine, putrescine, creatinine, and trimethylamine N-oxide). Further, studies of metabolites present during capacitation have shown that human spermatozoa secrete lactate, acetate, and malate, lactate being the main metabolite produced [41]. Lactate comes from the metabolization of ^13^ Cu-glucose and ^13^ Cu-fructose, indicating that human sperm produce ATP through glycolysis [11]. Carnitine and acetylcarnitine are related to the energy supply from lipid metabolism. In fact, these two molecules take part in β-oxidation, by regulating the transfer of fatty acids and acetyl donors into the mitochondria [23]. Amaral and colleagues [42] showed the importance of ATP production from lipid metabolism in human sperm in a study in which the incubation of ejaculates with etomoxir (an inhibitor of fatty acid β-oxidation -FAO-) was found to markedly reduce human sperm motility. Although the idea that human sperm motility is fueled by the oxidation of endogenous phospholipids was suggested a long time ago [24], these studies using NMR to detect sperm metabolites have shed some light on the energy sources of spermatozoa, and have been able to link sperm metabolites with human infertility [40,43,44].

### Metabolites for Diagnostic Tests of Human Infertility

Infertility is one of the major health issues around the world. In Europe, male infertility rates are between 10–15% and account for 50% of couples’ infertility problems [9]. The most frequent causes of male infertility range from genetic mutations to lifestyle, diseases or medications [45] Whereas the most frequent causes of female infertility are tubal diseases, idiopathic infertility, endometriosis and polycystic ovarian syndrome [46].

Most metabolomic studies of male infertility have focused on seminal plasma [47]. Seminal plasma consists of proteins, amino acids, enzymes, fructose and other carbohydrates, lipids and major minerals and trace elements (such as Zn ^2+^, Mg ^2+^, Ca ^2+^, K ^+^ and Na ^+^) secreted by the testes, epididymis, and accessory sex glands [48]. Therefore, some of the metabolites mentioned earlier could be candidates for being used as sperm quality markers [49]. Through ^1^ H-NMR metabolomics in human seminal plasma, lower levels of alanine, citrate and GPC, and higher levels of tyrosine, and phenylalanine have been related to oligozoospermia (decreased sperm number in the ejaculate) [50], while reduced levels of metabolites involved in phospholipid (choline), cholesterol and nucleoside metabolism and in the Krebs cycle, have been related to asthenozoospermia (decreased sperm motility) [51]. Moreover, teratozoospermia (an increase of sperm abnormalities) has been linked to greater levels of citric acid, choline, D-glucose, tyrosine, alanine, proline, leucine, lysine, myo-inositol, lactic acid, threonine, pyruvate, glutamine, valine and isoleucine, but with lower levels of glutamic acid, cholesterol and taurine [47]. To date, however, few studies have focused on sperm metabolites to elucidate the causes of human infertility. Reynolds and colleagues [40] performed ^1^ H-NMR in human sperm after density gradient centrifugation (DGC) and reported that sperm recovered from the interface showed greater levels of lactate, lipid, choline and GPC than those recovered from the pellet. To confirm whether the elevated lactate found in this sperm subpopulation was indicative of altered glycolysis, Calvert and colleagues [11] carried out ^13^ C-NMR in live human spermatozoa subjected to DGC and also observed increased levels of glycolysis in this subpopulation. An explanation for this could be the larger cytoplasm these cells have, as this is the cell compartment where glycolysis takes place [52]. Another possibility is that the sperm from the pellet, which shows improved morphology and motility, tightly controls its energy production to limit ROS levels and, therefore, avoid DNA damage [53]. Collectively, these interesting findings correlate the metabolomic profile of spermatozoa to their morphology and/or motility, suggesting that sperm metabolites could be potential biomarkers of male fertility and therefore, that NMR could be of great interest for diagnostic purposes. Still, more studies need to be performed to establish a list of sperm metabolites that can be widely used as biomarkers of male fertility.

## 4. NMR and Bull Fertility

As a main source of animal protein, livestock rearing plays an important role in global food systems. Currently, cattle production for the beef industry in the European Union occupies the third position in the world [54]. In this context, bull fertility is important, as a decrease in fertility affects the conception rate resulting in diminished production and, therefore, lower economic returns at the farm level [13]. Conventional methods available to predict bull fertility are still of limited use. The study of the sperm functional genome including transcriptome, proteome, and metabolome could be useful to predict bull fertility. Through metabolomic approaches, specific metabolites have been identified in both seminal plasma and sperm [55].

Characterization of the metabolic profile of bull seminal plasma by NMR could be useful to distinguish between bulls of high and low fertility [48]. Kumar and colleagues [48] showed that high-fertility bulls had lower levels of citrate and isoleucine versus higher levels of tryptamine, taurine, and leucine in seminal plasma. However, Velho and colleagues [13] reported that high fertility bulls had more fructose and less oxoglutaric acid, ornithine, L-leucine and D-manitol in seminal plasma determined by GC–MS. It seems that cryopreservation can alter the seminal plasma metabolome. The fresh seminal plasma of high fertility bulls was analyzed using LC–MS and results showed a different lipid profile (increased levels of l-acetylcarnitine, glycerol tripropanoate, 2,3-diacetoxypropyl stearate and GPC, and a decrease of lysoPC (p-16:0) and butyrylcarnitine) compared to low fertility bulls [35]. Therefore, the presence in seminal plasma of some metabolites can be associated with bull fertility and thus could be useful biomarkers [48].

Besides seminal plasma, bull spermatozoa has been also examined through metabolomics. In 1987, Robitaille et al. [56] reported that P-NMR on samples of bull spermatozoa revealed the presence of phosphomonoesters, GPC, but more notably free nucleotide diphosphate (NDP) and triphosphates (NTP). Since then, 22 metabolites have been structurally identified by GC–MS in Holstein bull spermatozoa showing different levels of field fertility [55]. Sperm metabolome analysis in this species has revealed that the majority of metabolites present are organic acids and derivatives, and fatty acids and conjugates [12]. Saraf and colleagues [57] also reported, through an LC–MS/MS metabolomic procedure, that some metabolites present in spermatozoa could be used to differentiate between highly fertile crossbred bulls (spermine, L-cysteine) or lowly fertile bulls (dihydrolipoamide, cysteinyl leukotriene and inositol 1,4,5 trisphosphate). This group described that some metabolites (hypotaurine, selenocysteine, L malic acid, D cysteine and chondroitin) were differentially expressed, suggesting they could be metabolomic targets for the assessment of bull sperm fertility [57]. However, in another study in which an LC–MS metabolomic approach was used on frozen bull semen, lower concentrations of metabolites, such as leucine, and higher concentrations of glutamic acid and cysteine were observed in spermatozoa from low- compared to high fertility bulls [58]. Overall, these findings highlight NMR and other metabolomic approaches as an interesting tool to predict male fertility in this species.

## 5. NMR and Mammalian Sperm Metabolites

**Figure 1:**
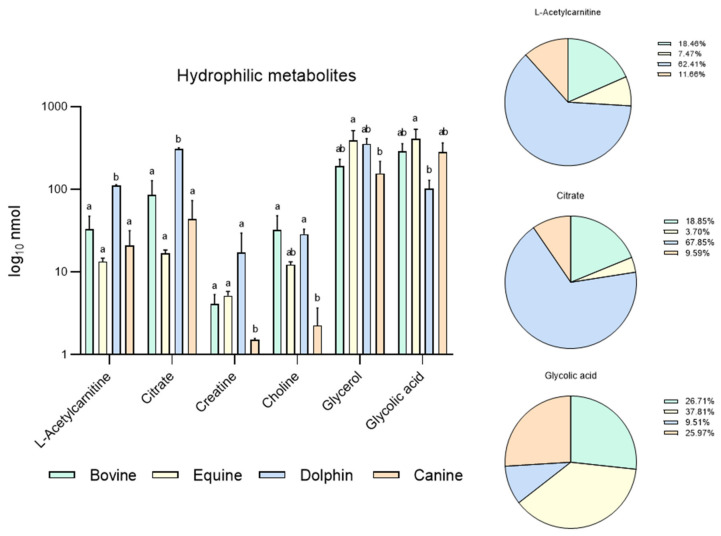
Metabolites identified in bovine, equine, dolphin and canine sperm showing differences between species in 16 metabolites identified in the hydrophilic phase of sperm extracts by NMR spectroscopy. ^a,b^ Different letters indicate significant differences (*p* < 0.05) among species for each metabolite. Bovine and dolphin data were already published [60], equine and canine data belong to this new experiment.

**Figure 2:**
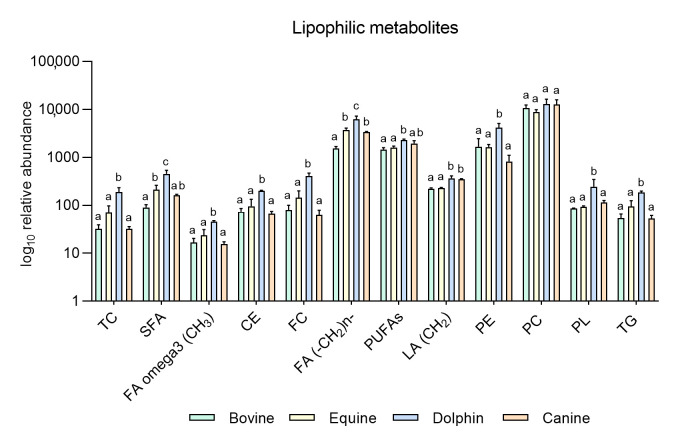
Metabolites identified in the lipophilic phase of bovine, equine, dolphin and canine sperm extracts by NMR spectroscopy. ^a,b,c^ Different letters indicate significant differences (*p* < 0.05) among species for each metabolite. Total cholesterol (TC), saturated fatty acids (SFA), fatty acids omega 3 (CH_3_) (FA omega3), cholesterol ester (CE), free cholesterol (FC), fatty acids (-CH_2_)n- [FA (-CH_2_)n-], polyunsaturated fatty acids (PUFAs), linoleic acid (CH_2_) [(LA(CH_2_)], phosphatidylethanolamine (PE), phosphatidylcholine (PC), phospholipids (PL), triglycerides (TG). Bovine and dolphin data were already published [60], equine and canine data belong to this new experiment.

NMR could also be a very useful approach to analyze both sperm lipid contents and sperm metabolism. Recently, through sperm hydrophilic and lipophilic metabolome extraction, we examined differences in metabolites between bull and dolphin, as they are two species of the same order but with different diets: herbivores and carnivores, respectively [59]. Our results indicated differences in six hydrophilic metabolites (higher expression of four and lower expression of two in dolphin sperm, Figure 1) and 11 lipophilic metabolites (all showing higher expression in dolphin sperm) (Figure 2). We also noted that dolphin sperm cells maintained their normal motility for more than 3 days without glucose and pyruvate supplementation, contrasting with the situation in mice and bull sperm which stop moving after 1 and 3 h, respectively [60]. However, when dolphin sperm were exposed to etomoxir (an inhibitor of carnitine palmitoyl-transferase 1 a and of mitochondrial fatty acid β-oxidation), cell motility dropped after only 1 h of incubation, indicating the complete undoing of the glycolytic pathway while supporting a central role of β-oxidation in ATP generation to fuel normal dolphin sperm motility. Etomoxir-dependent β-oxidation inhibition has been shown to reduce sperm motility in various species including humans [23] and boar [51].

Based on these results, our group conducted a new experiment including new data in two other species: equine and canine. It was designed to compare endogenous sperm metabolites among herbivorous: horse and cow [59], carnivorous (dolphin, [59]), and omnivorous mammals (dog). Sperm individual samples of the four species, dog (*n* = 3), cow (*n* = 3), dolphin (*n* = 2) and horse (*n* = 3), were collected using an artificial vagina from normozoospermic males showing normal sperm counts and sperm morphology (<30% abnormal sperm cells). Semen collection was performed according to institutional and European regulations. Sperm preparation, purification, NMR extraction and measurements were performed as described above. Results are displayed in Figure 1 and Figure 2. Sixteen metabolites were identified in the hydrophilic phase (butyric acid, 3-hydroxyisobutyicate, propylene glycol, isobutyric acid, lactate, putrescine, acetic acid, L-acetylcarnitine, glutamic acid, citrate, creatinine, choline, glycolic acid, creatinine and glycerophosphocholine) and levels of 6 of these metabolites (L-acetyl carnitine, citrate, creatinine, choline, glycerol and glycolic acid) differed among species (Figure 1). In the lipophilic phase, we identified 12 metabolites: total cholesterol (TC), saturated fatty acids (SFA), fatty acids omega 3 CH3 (FA omega3), cholesterol ester (CE), free cholesterol (FC), fatty acids (-CH2)n- [FA (-CH2)n-], polyunsaturated fatty acids (PUFAs), linoleic acid (CH2) [(LA(CH2)], phosphatidylethanolamine (PE), phosphatidylcholine (PC), phospholipids (PL) and triglycerides (TG); (Figure 1 and Figure 2). Higher quantities of all the lipophilic metabolites were detected in the dolphin than the herbivores and omnivores (Figure 2).

These results support the notion that these substances are substrates for energy production in sperm cells. Interestingly, horses appear to have higher levels of lipophilic metabolites compared to cattle. Some differences have been already described in livestock species. Hence, while OXPHOS is the predominant pathway to obtain energy in bull sperm, ram spermatozoa can fuel their motility via both glycolysis and OXPHOS [61]. In the hydrophilic phase of sperm samples from the bottlenose dolphin, quantities of L-acetylcarnitine and citrate were one hundred-fold those observed in the other three species. These metabolites help transport fatty acids into mitochondria for their β-oxidation and the Krebs cycle respectively, contributing to the hypothesis of fatty acid catabolism as the source of sperm energy in this species. Similarly, putreiscine, acetic acid, glutamic acid, creatine and glycerol contents were also higher in the dolphin sperm (Figure 1). The herbivores examined showed similar patterns except for L-acetylcarnitine, citrate and choline which appeared in higher amounts in the cow than horse sperm cells. The presence of L-acetylcarnitine in spermatozoa has been related to good sperm motility [62] and fertility level due to its antioxidant functions [63]. In addition, it has been reported that seminal plasma from bulls of high fertility show higher levels of L-acetylcarnitine than low fertility bulls [35]. Moreover, bull epididymal sperm incubated in the absence of substrates have been reported to use endogenous L-acetylcarnitine to produce energy for motility [64]. This determines that this compound could be a candidate as a male fertility biomarker, at least in this species. In contrast, the last two metabolites mentioned appeared in the much lower measure in the sperm from the strict carnivores and herbivores examined here. These findings indicate dramatic sperm energetics modifications in carnivores, especially appreciable in Odontoceti species, towards fatty acid and ketone body catabolism.

## 6. Conclusions

This review provides insight into the use of NMR to analyze the metabolome of sperm and seminal plasma and its association with sperm functions. The NMR technique could be a promising screening tool to detect biomarkers of male infertility when the cause of infertility is unclear, to predict animal fertility, and to define new media for improving sperm motility, capacitation and/or in vitro fertilization.

## Data Availability

Not applicable.
